# Seaweed-Derived Iodine Intake During the Korean Postpartum Period: A 1-Year Follow-Up Study

**DOI:** 10.3390/healthcare14030298

**Published:** 2026-01-24

**Authors:** Jihee Choi, Se-A Lee, Na Young Yoon, Hae-Jeung Lee

**Affiliations:** 1 Department of Food and Nutrition, College of Bionanotechnology, Gachon University, Seongnam-si 13120, Republic of Korea; jiheechoi@gachon.ac.kr (J.C.); dltpdk1104@gachon.ac.kr (S.-A.L.); 2 Institute for Aging and Clinical Nutrition Research, Gachon University, Seongnam-si 13120, Republic of Korea; 3 Food Safety and Processing Research Division, National Institute of Fisheries Science, Busan 46083, Republic of Korea; dbssud@korea.kr

**Keywords:** iodine, seaweed, postpartum, thyroid hormone, follow-up study, dietary assessment

## Abstract

**Highlights:**

**What are the main findings?**

- In this small follow-up study, no thyroid disease was observed at one year.
- Abnormal T3, FT4, and TSH levels did not differ by iodine-intake quartiles.
- Findings are preliminary due to limited sample size, high dropout, and restricted hormonal markers.

**What are the implications of the main findings?**

- Results do not show a clear association between short-term postpartum iodine intake and thyroid dysfunction but cannot exclude potential risks.
- Provides initial data for an understudied population.
- Larger longitudinal studies are required to confirm these observations.

**Abstract:**

**Background:** Seaweed consumption is a major source of dietary iodine in Korea, particularly among lactating women during the postpartum period. This practice raises concerns regarding short-term iodine excess and its potential effects on thyroid function. We examined the prevalence of thyroid disease and hormone abnormalities 1 year after childbirth among postpartum women with varying levels of seaweed-derived iodine intake. **Methods:** Between 17 July 2021 and 10 December 2021, 147 postpartum women were enrolled within two weeks after childbirth at postpartum care centers in Korea, which provide structured residential maternal and infant care, including standardized meals, during the early postpartum period. Participants provided informed consent and completed baseline questionnaires and dietary assessments. Iodine intake, including seaweed soup consumption during the 8-week postpartum period, and infant growth indicators were evaluated. A total of 81 participants completed the 1-year follow-up. At follow-up, dietary records, thyroid disease prevalence, hormone levels, urinary iodine concentration, and infant growth indicators were assessed. **Results:** At 1 year, none of the 81 participants had thyroid disease. The prevalence of abnormal triiodothyronine (T3), free thyroxine (FT4), and thyroid-stimulating hormone (TSH) was analyzed by iodine-intake quartiles, revealing no significant differences (T3: *p* = 0.4175; FT4: *p* = 0.1591; TSH: *p* = 0.9344). **Conclusions:** These findings suggest that the evidence regarding an association between short-term postpartum iodine intake and thyroid outcomes one year after childbirth remains inconclusive. Owing to the limited sample size, high attrition, and observational design, the study may have been underpowered to detect clinically meaningful differences, and potential effects cannot be excluded. Therefore, these results should be interpreted cautiously, and larger, well-designed longitudinal studies with repeated thyroid assessments are needed to better clarify the long-term implications of postpartum iodine exposure.

## 1. Introduction

Iodine is an essential micronutrient for thyroid hormone synthesis, and a healthy adult body contains approximately 15–20 mg of iodine, 70–80% of which is concentrated in the thyroid gland [1]. Iodine deficiency can lead to thyroid hypertrophy, hypothyroidism, congenital malformations in the fetus, and delayed physical development in children and adolescents [2]. Globally, iodine deficiency remains a major public health concern, particularly among pregnant women and women of childbearing age, as it represents the leading preventable cause of intellectual disability in children [3]. While excess iodine can also lead to hypothyroidism [4], iodine deficiency has posed a greater population-level risk worldwide [2].

The primary food sources of iodine include iodine-fortified salt, bread, and seaweed, among others [5]. Owing to their geographical characteristics, Koreans traditionally consume an iodine-rich diet, with approximately 65.6% of their iodine intake coming from seaweed [6]. Unlike many countries with mandatory universal salt iodization programs, Korea does not enforce compulsory salt iodization; consequently, iodine intake in the Korean population is primarily derived from naturally iodine-rich foods rather than fortified salt. Seaweed and seaweed-based products therefore remain major contributors to iodine intake, resulting in relatively high and variable iodine exposure in certain population groups, including postpartum women.

Traditionally, Korean women consume brown seaweed (*Undaria pinnatifida*) soup during the postpartum period, a practice believed to promote maternal recovery but one that substantially increases iodine intake among lactating women [7]. This intake was significantly higher in Korean lactating women than in their counterparts from other countries [7], and their early postpartum intake (within 2–5 days after childbirth) exceeded the upper intake level of 2400 μg/day recommended for Korean adults [8]. A study reported that Korean lactating women with excessive iodine intake have high breast milk iodine concentrations, which may increase the risk of hypothyroidism in vulnerable infants, particularly those born preterm [9].

Despite these concerns, evidence regarding whether short-term excessive iodine intake during the postpartum period has measurable long-term thyroidal consequences remains limited. Most existing studies have focused on iodine deficiency or chronic iodine excess, while few have examined whether temporary iodine excess during early postpartum life is associated with subsequent thyroid dysfunction in otherwise healthy women.

Therefore, the present study aimed to explore whether temporary excessive iodine intake from seaweed consumption during the postpartum period is associated with thyroid outcomes one year after childbirth in otherwise healthy Korean women.

## 2. Materials and Methods

### 2.1. Study Design and Population

This study involved a follow-up study designed to evaluate the association between iodine intake and thyroid disease in postpartum women. The required sample size was calculated based on the following hypothesis [10]. We estimated a minimum sample size using power calculations with a confidence interval of 95%, aiming for a power of 0.80 (80%) and an α error probability of 0.05. The required sample size was determined to be 117 participants using G-power software (version 3.1.9.4; Heinrich Heine University Düsseldorf, Düsseldorf, Germany). Accounting for a 20% loss to follow-up, we aimed to recruit 147 participants.

To recruit participants, promotional posters were distributed and displayed at postpartum care centers in Seoul, Gyeonggi, and Incheon, Korea. In Korea, postpartum care centers are specialized residential facilities where mothers and newborns stay for approximately 1–2 weeks after childbirth to receive medical monitoring, standardized meals, and infant care support. This setting allowed systematic assessment of dietary intake during the early postpartum period. Participants were included if they met the following criteria: postpartum women within 2 weeks of giving birth who agreed to participate in the study and signed a consent form. For newborns, legal representatives were required to provide written informed consent for their participation. Participants with (1) a history of thyroidectomy and current use of thyroid medications for thyroid disease and/or (2) a history of hyperthyroidism, hypothyroidism, thyroid neoplasm, thyroiditis, or any other severe comorbidities were excluded.

**Figure 1:**
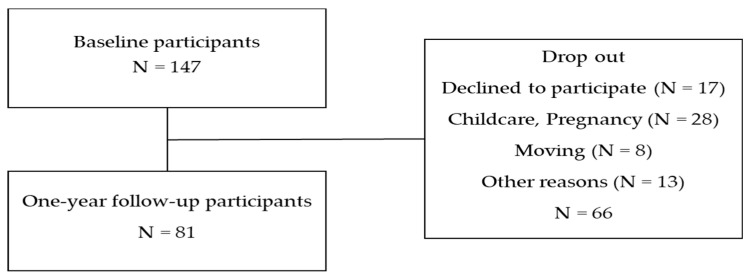
Flow diagram of study participants. Postpartum women were recruited within two weeks after childbirth. A total of 147 postpartum women without a history of thyroid disease were enrolled at baseline. Of these, 81 women completed the clinical and dietary assessments at the 1-year follow-up.

**Figure 2:**
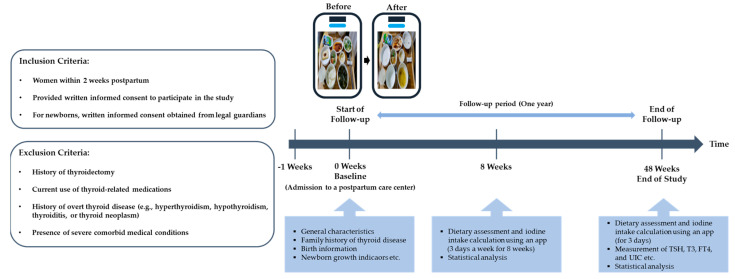
Study design and timeline. Overview of the study design, including participant recruitment during the early postpartum period, assessment of dietary iodine intake during the 8-week postpartum period, and evaluation of thyroid-related outcomes at 1 year after childbirth.

A total of 147 women were enrolled in this study. Among them, 81 completed clinical and dietary assessments 1 year after childbirth during follow-up (baseline at early postpartum: 17 July 2021 to 10 December 2021; follow-up at 1 year after childbirth: 17 July 2022 to 10 December 2022). Infant height and weight data were collected from hospital birth records provided by the participants. Figure 1 presents a flow diagram of participant selection. Figure 2 illustrates the study design. Patients or members of the public were not involved in the design, conduct, reporting, or dissemination plans of this research.

This study was approved by the Gachon University Institutional Review Board on 15 July 2021 (IRB, 1044396-202007-HR-136-02) and performed according to the ethical standards of the Declaration of Helsinki as revised in 2013. After receiving explanations of the study’s goals, all participants provided written informed consent. The study protocol was registered with the Clinical Research Information Service (https://cris.nih.go.kr/cris/index/index.do (accessed on 6 November 2023), KCT0008929), and protocol modifications were publicly reflected through CRIS.

### 2.2. Dietary Assessment and Iodine Intake Calculation

#### 2.2.1. Baseline

To calculate iodine intake during the 8-week postpartum care period, we collected weekly dietary records for 3 days (two weekdays and one weekend) as well as data on the weekly intake of brown seaweed soup over 3 days (two weekdays and one weekend). This early postpartum period was selected a priori as the primary exposure window because iodine intake from traditional postpartum diets is markedly elevated during this time and represents a distinct, short-term exposure relevant to subsequent thyroid outcomes. During their stay in the postpartum care center, to ensure accurate dietary records, we obtained the menu of each postpartum care center in advance. Participants were then asked to photograph their food before and after consumption for 3 days each week over the 8-week period and record this information using a mobile application. Professional nutritionists analyzed these photographs to estimate food waste and thereby refine the intake estimates for brown seaweed soup. All photographs were independently reviewed by two trained nutritionists using a standardized protocol to estimate actual intake and plate waste; discrepancies were resolved by consensus [11]. Nutrient intake—based on the 3-day dietary records—was calculated using the Computer Aided Nutritional Analyses Program (CAN-Pro) (version 5.0; The Korean Nutrition Society, Seoul, Republic of Korea). Nutrient intake was calculated using the CAN-Pro dietary analysis program, which is based on the Korean food composition database and includes iodine values from foods and seasonings, including salt, thereby allowing iodine intake from salt consumption to be incorporated into total dietary iodine estimates. As for the iodine content of the dried brown seaweed (*Undaria pinnatifida*), we used 15,800 μg per 100 g from the preliminary analysis of 30 samples [11]. Brown seaweed soup intake was standardized by classifying the six types of bowls commonly used by early postpartum women. A bowl containing 250 g of brown seaweed soup prepared with 7 g of dried seaweed was classified as one portion (100%), while portions prepared with lesser amounts of dried seaweed were classified as 70% or 50%. Brown seaweed soup intake at each meal was calculated by subtracting leftovers from the served amount. To calculate total daily iodine intake, iodine derived from dietary supplements was assessed and added to dietary iodine intake estimates.

#### 2.2.2. Follow-Up at 1 Year After Childbirth

Dietary records for 3 days (two weekdays and one weekend) were collected prior to urine collection. To ensure accurate dietary records, participants were asked to record all meals consumed and photograph their food before and after consumption using a mobile application, following the same method used at baseline. Brown seaweed soup and nutrient intake were calculated using the same method as at baseline. In addition, a food frequency questionnaire (FFQ) was used to assess the frequency of seaweed consumption during the first postpartum year.

### 2.3. T3, FT4, TSH, and UIC Measurements

TSH, T3, FT4, and spot UIC levels were assessed only in postpartum women at the 1-year follow-up. As spot UIC reflects recent iodine intake and exhibits substantial intra-individual variability, it was used to characterize iodine status at follow-up rather than to retrospectively quantify iodine exposure during the early postpartum period. Measurements were not conducted in infants or children. For thyroid evaluation, blood samples were collected and stored in a freezer (−70 °C) for analysis. Serum levels of TSH, T3, and FT4 were measured using a chemiluminescent immunoassay. For the assessment of iodine status, morning spot urine samples were collected at the 1-year follow-up, stored in a freezer, and analyzed using an inductively coupled plasma mass spectrometry device (ICP-MS) (PerkinElmer NexION series; PerkinElmer Inc., Waltham, MA, USA).

### 2.4. Statistical Analysis

Continuous variables are expressed as the mean ± standard error (SE), and categorical variables are presented as frequencies with percentages. Group differences in continuous variables were assessed using Student’s t-test or one-way analysis of variance (ANOVA), as appropriate. Categorical variables were compared using the Chi-squared test, with Fisher’s exact test applied when expected cell counts were <5. In addition, the prevalence of thyroid hormone abnormalities was compared across iodine-intake quartiles using Fisher’s exact test. Given the very small number of abnormal outcomes, regression-based models were not applied because stable model estimation was not feasible. Total dietary iodine intake during the early postpartum period (8 weeks after childbirth), including iodine derived from brown seaweed soup and dietary supplements, was categorized into quartiles based on its distribution among participants and used as the primary exposure variable. In addition, total iodine intake at the 1-year follow-up was also categorized into quartiles and examined in relation to thyroid hormone levels measured at the same time point as a secondary, cross-sectional analysis. These analyses were considered exploratory and intended to assess concurrent associations rather than causal effects of postpartum iodine exposure. To account for potential confounding, multivariable analyses were performed with adjustment for maternal age, body mass index (BMI), parity, breastfeeding status, and total energy intake. Because iodine intake from dietary supplements was incorporated into total iodine intake estimates, supplement use was not analyzed as a separate exposure variable. All tests were two-sided, with statistical significance defined as *p* < 0.05. Statistical analyses were conducted using SAS software (version 9.4; SAS Institute Inc., Cary, NC, USA) and R software (version 3.6.1; R Foundation for Statistical Computing, Vienna, Austria).

## 3. Results

### 3.1. Participants’ General Characteristics

We compared the participants’ baseline characteristics with those after 1 year of follow-up (Table 1 and Table S1). No significant differences in the participants’ general characteristics or newborn growth indicators were observed between baseline and 1-year follow-up. In addition, baseline characteristics were compared between participants who completed the 1-year follow-up (retained participants, *n* = 81) and those who withdrew during follow-up (withdrawn participants, *n* = 66). No statistically significant differences were observed between the two groups for demographic, reproductive, or dietary variables, except for family history of thyroid disease, which was more frequently reported among retained participants (Supplementary Table S2). Given the small absolute number of affected individuals, this difference is unlikely to have materially influenced the study findings.

### 3.2. Nutrient Intake During the Postpartum Period and at 1-Year Follow-Up

The participants’ nutrient intake during the postpartum period and 1-year follow-up is detailed in Supplementary Table S3. Daily energy, carbohydrate, fat, protein, fiber, vitamin, calcium, and iron intake during the postpartum period were significantly higher than those at the 1-year follow-up, as participants received nutrients through the diet provided at the postpartum care center. Iodine intake during the postpartum period was significantly higher than that at the 1-year follow-up (*p* < 0.0001). In addition, the frequency of seaweed consumption during the first postpartum year, assessed using a food frequency questionnaire, did not differ significantly from seaweed intake estimated from the 3-day dietary records collected at the 1-year follow-up.

### 3.3. Total Iodine Intake and Iodine Intake from Brown Seaweed Soup During the Postpartum Period

The results of dietary assessments, total iodine intake, and iodine intake from brown seaweed soup during the postpartum period are presented in the Supplementary Table S4. Total daily iodine intake peaked during the first and second weeks of the postpartum period and gradually decreased from the third week onward. The Recommended Nutrient Intake (RNI) for lactating women is set at 340 μg/day, without establishing a Tolerable Upper Intake Level (UL). In contrast, the UL for adults aged >19 years has been set at 2400 μg/day. During the postpartum period, the study participants’ total iodine intake exceeded the UL for adults only in week 1. Furthermore, the RNI was surpassed even in week 8, when total iodine intake was at its lowest during the postpartum period.

### 3.4. Prevalence Rates of Abnormal T3, FT4, and TSH Levels According to Iodine Intake During the Postpartum Period

We determined the prevalence rates of thyroid disease and abnormal hormone levels (TSH, T3, and FT4) among the participants at 1-year follow-up as well as those of congenital hypothyroidism in newborns and thyroid disease in infants after 1 year. None of the 81 participants had thyroid disease. Because no cases of thyroid disease occurred during the follow-up period, estimation of relative risk was not feasible. None of the newborns presented with congenital hypothyroidism, and no infants were diagnosed with thyroid disease 1 year later.

The rates of participants with abnormal T3, FT4, and TSH levels were calculated for each quartile group according to total iodine intake, including that from brown seaweed soup consumption, during the postpartum period. The rates of abnormal T3 levels were 3 (15%), 1 (5%), 5 (23.8%), and 3 (15%) for Q1, Q2, Q3, and Q4, respectively, exhibiting no significant differences among the four groups (*p* = 0.4175). The rates of abnormal FT4 levels were 1 (5%), 0 (0%), 2 (9.5%), and 4 (20%) for the respective quartiles, with no significant differences among the four groups (*p* = 0.1591). Similarly, the rates of abnormal TSH levels yielded no significant differences among the groups (*p* = 0.9344), with results of 1 (5%), 2 (10%), 1 (4.8%), and 1 (5%) for the respective quartiles (Table 2).

### 3.5. Prevalence Rates of Abnormal T3, FT4, and TSH Levels According to Iodine Intake at 1-Year Follow-Up

At the 1-year follow-up, none of the 81 participants who completed the study were diagnosed with thyroid disease. The rates of participants with abnormal T3, FT4, and TSH levels were calculated for each quartile group according to total iodine intake, including that from brown seaweed soup consumption. The rates of abnormal T3 levels were 3 (15%), 3 (15%), 4 (19.1%), and 2 (10%) for Q1, Q2, Q3, and Q4, respectively, indicating no significant differences among the four groups (*p* = 0.9721). The rates of abnormal FT4 levels were 1 (5%), 1 (5%), 5 (23.8%), and 0 (0%), respectively, with no significant differences among the four groups (*p* = 0.0589). Likewise, no significant differences in the rates of abnormal TSH levels (1 [5%], 0 [0%], 2 [9.5%], and 2 [10%], respectively) were noted among the four groups (*p* = 0.7476; Table 3). In addition, exploratory analyses treating thyroid hormone levels (T3, FT4, and TSH) as continuous variables did not reveal significant associations with iodine intake.

### 3.6. Iodine Intake and UIC at 1-Year Follow-Up

The single urine iodine excretion and daily total iodine intake, including that from brown seaweed soup consumption, of women at the 1-year follow-up (Table 4). The average and median iodine intakes were 220.5 ± 65.5 and 60.9 μg/day, respectively. The average and median UIC values were 423.1 ± 88.2 and 194.6 μg/L, respectively. According to the WHO, iodine intake is considered adequate when the median UIC among adults is 100–199 μg/L [12]. Accordingly, the iodine intake of the participants at 1-year follow-up was found to be adequate.

### 3.7. Correlation of Iodine Intake with Thyroid Hormones

**Figure 3:**
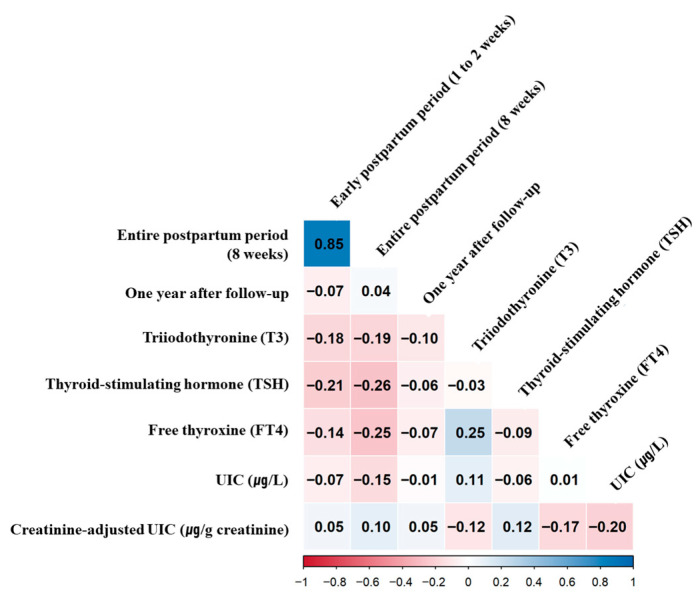
Correlations between iodine intake and thyroid-related biomarkers. Spearman’s correlation analysis was performed to examine the associations between iodine intake variables (early postpartum, 8-week postpartum, and 1-year follow-up) and thyroid-related biomarkers measured at 1 year, including triiodothyronine (T3), free thyroxine (FT4), thyroid-stimulating hormone (TSH), and urinary iodine concentration (UIC). Statistical significance is indicated as follows: *p* < 0.05.

Spearman’s correlation analysis showed a strong positive correlation between iodine intake at 1–2 weeks and at 8 weeks postpartum (ρ = 0.85, *p* < 0.0001). Iodine intake during the 8-week postpartum period was negatively correlated with TSH (ρ = −0.26, *p* < 0.05) and FT4 (ρ = −0.25, *p* < 0.05), but not with T3. No significant correlations were observed between iodine intake at 1–2 weeks postpartum and thyroid hormone levels. A positive correlation was noted between T3 and FT4 (ρ = 0.25, *p* < 0.05). At the 1-year follow-up, iodine intake was not significantly associated with T3, FT4, or TSH. Additionally, no significant correlations were found between urinary iodine indicators, including spot urinary iodine concentration (UIC) and creatinine-adjusted UIC, and either iodine intake variables or thyroid hormone levels. These results remained consistent after adjusting for potential confounders, including maternal age, BMI, parity, breastfeeding status, and energy intake, as shown in Figure 3.

## 4. Discussion

This follow-up study examined the association between temporary iodine excess during the early postpartum period and thyroid outcomes one year after childbirth. Overall, no statistically significant differences were observed in the prevalence of thyroid disease or abnormalities in T3, FT4, and TSH levels according to quartiles of seaweed-derived iodine intake. However, these findings should not be interpreted as evidence of the absence of an effect.

Given the limited sample size, the small number of abnormal thyroid hormone outcomes, and the observational nature of the study, the statistical power to detect clinically meaningful differences was restricted. Consequently, small to moderate associations between postpartum iodine intake and later thyroid function cannot be excluded. The present results should therefore be regarded as inconclusive and interpreted cautiously, underscoring the need for larger longitudinal cohort studies with repeated thyroid assessments to better characterize potential long-term risks.

A previous cohort study of Korean pregnant women found that dietary iodine-rich intake during pregnancy did not affect maternal thyroid function or birth outcomes [13]. Another prospective cohort study examining the association between iodine intake and thyroid disease in Korean postpartum women found that high iodine intake did not affect the occurrence of thyroiditis in healthy women [14]. However, since that time, no other cohort study has sought to verify the association between dietary iodine intake and thyroid disease in Korean postpartum women.

This study’s results indicated that the average dietary iodine intake of postpartum women was 1159.0 ± 86.2 µg/day during the postpartum period (8 weeks) (Supplementary Table S4), yielding a value within the UL of 1100 µg/day established by the American Institute of Medicine for adults aged ≥18 years from the fourth week [15,16] and that of 220.5 ± 65.5 µg/day after 1 year of follow-up.

These results indicate that the dietary iodine intake of postpartum women is decreased compared to the results of our previous study [11]. The reasons for the decrease are presumed to be as follows: First, the intake of brown seaweed soup decreased among postpartum women, likely due to increased awareness of the potential adverse health effects of excessive iodine intake. Second, in our previous study [11], broth extracted from dried kelp (iodine content: 192,700 µg/100 g) was more often used in brown seaweed soup than in the present study, which substantially increased the average iodine content of the soup. Therefore, dietary iodine intake during the early postpartum period was conceptualized as a transient exposure, whereas thyroid hormone outcomes assessed at one year were considered potential downstream effects of this short-term excess rather than reflections of concurrent iodine status.

In several Western settings, iodine insufficiency among lactating women has been reported, often related to low intake of iodine-rich foods and limited supplement use. For example, a Swedish study suggested mildly inadequate iodine intake among lactating women, particularly among non-iodine supplement users [17].

In a study on the association between iodine intake and thyroid disease in older participants from Iceland and Denmark, iodine deficiency was associated with hyperthyroidism, while excess iodine intake increased serum TSH levels and was linked to hypothyroidism [18]. However, this trend typically occurs in individuals with some degree of thyroid autoimmunity [19], and thyroid disease is more likely to arise when iodine intake rapidly increases without control in areas of moderate iodine deficiency [20].

In contrast, in iodine-replete seaweed-consuming populations (e.g., Korea and Japan), habitual iodine exposure can be high and highly variable, largely driven by seaweed and seaweed-based products [21].

This cross-country contrast highlights the relevance of our study: while many populations are primarily concerned with iodine deficiency during lactation, seaweed-consuming populations must also consider transient iodine excess. Importantly, iodine excess is more likely to cause clinically relevant thyroid dysfunction in susceptible individuals (e.g., underlying thyroid autoimmunity), whereas healthy individuals may adapt through autoregulatory mechanisms [21].

For most individuals, temporary excessive iodine intake does not lead to disease because several mechanisms in the body can mitigate the adverse effects of excessive iodine intake [20,21,22,23,24].

Several autoregulatory mechanisms have been proposed to explain why transient excessive iodine intake does not necessarily result in sustained thyroid dysfunction in healthy individuals. One such mechanism involves the sodium–iodide symporter (NIS), which mediates active iodide transport in thyroid and non-thyroid tissues and contributes to the regulation of intracellular iodine concentrations under conditions of iodine excess [22,23]. Another well-described mechanism is the acute Wolff–Chaikoff effect, a transient reduction in thyroid hormone synthesis following exposure to high iodine levels, which has been primarily demonstrated in experimental animal models [25]. In these models, normal thyroid hormone synthesis typically resumes within 24–48 h despite continued iodine exposure, a process referred to as escape or adaptation from the acute Wolff–Chaikoff effect [26]. This adaptive response has been attributed, at least in part, to reduced iodide transport into the thyroid gland, potentially mediated by downregulation of NIS activity [27,28]. However, escape from the Wolff–Chaikoff effect does not occur uniformly across all individuals. Susceptible populations, including those with underlying autoimmune thyroid disease such as Hashimoto’s thyroiditis or Graves’ disease, may fail to adapt and are therefore at increased risk of iodine-induced hypothyroidism, which can be transient or permanent [29,30].

Because our study population consisted of otherwise healthy postpartum women without known thyroid disease, these autoregulatory mechanisms may partly explain the absence of overt thyroid dysfunction observed at one year of follow-up. Nevertheless, given the observational design and limited outcome assessment, these mechanistic considerations should be interpreted as contextual rather than causal explanations.

In addition, dietary factors such as cruciferous vegetable intake, which contains goitrogenic compounds, may interfere with iodine utilization and thyroid hormone synthesis [31,32]. Although Table 1 presents the frequency of cruciferous vegetable consumption among participants during the postpartum period, the specific intake thresholds at which these foods meaningfully affect iodine metabolism remain unclear, and their potential influence in the present study could not be quantified.

This study has several limitations that should be acknowledged. First, because iodine exposure during the postpartum period and thyroid outcomes were assessed at different time points, misclassification of biologically relevant exposure is possible, and associations between short-term iodine excess and later thyroid function may have been attenuated. Second, regarding the sampling strategy, when estimating the required sample size, we were unable to identify a previous study with an identical design; therefore, the sample size calculation was based on the most comparable available study. Although an a priori power calculation was performed and the recruitment target was adjusted to account for anticipated attrition, the final sample size was reduced due to loss to follow-up. Consequently, the representativeness and generalizability of the findings may be limited. Third, the proportion of participants lost to follow-up was higher than expected. Although participant withdrawal occurred in accordance with ethical principles respecting autonomy, this attrition may have introduced selection bias and reduced the statistical power of the study. As a result, the study may not have been sufficiently powered to detect small or moderate differences in thyroid hormone abnormalities, particularly given the low number of abnormal cases within each iodine-intake quartile. Fourth, thyroid function was assessed only once at the 1-year follow-up using serum T3, FT4, and TSH levels. This single time-point assessment may have failed to capture transient postpartum thyroid dysfunction, including postpartum thyroiditis, which often occurs within the first year after childbirth. In addition, thyroid autoantibodies were not measured; therefore, autoimmune thyroid disease could not be identified. As a result, outcome misclassification is possible, and the true association between postpartum iodine exposure and thyroid dysfunction may have been underestimated. Because thyroid dysfunction after pregnancy can be transient and heterogeneous in timing, the lack of serial thyroid measurements limits the ability to fully characterize temporal patterns of iodine-related thyroid responses in this population. Fifth, the small number of participants within each iodine-intake quartile limited the robustness of statistical analyses and further constrained the ability to detect clinically meaningful differences across exposure groups. In particular, the reliance on categorical comparisons across quartiles, combined with the low number of abnormal outcomes, may have increased the risk of type II error; therefore, the absence of statistically significant findings should not be interpreted as evidence of no association. Sixth, although individuals with a known history of thyroidectomy, hyperthyroidism, hypothyroidism, thyroid neoplasm, thyroiditis, current thyroid medication use, or severe comorbidities were excluded, undiagnosed autoimmune thyroiditis or subclinical thyroid disease could not be completely ruled out because exclusions were based on self-reported medical history. Finally, correlation analyses should be interpreted with caution, as the observed coefficients were modest, the sample size was limited, and residual confounding may persist despite statistical adjustment. Taken together, while the findings provide preliminary insights into the potential long-term effects of postpartum iodine intake, they should be interpreted carefully within the context of these methodological limitations.

Despite these limitations, this study has certain strengths. First, as few follow-up studies have investigated the risk of hormone level deviation from the normal range according to iodine intake during the postpartum period, it adds valuable data to the field. Second, we classified bowls of brown seaweed soup into six types and divided their density into three stages to obtain a more accurate assessment of intake among postpartum women. Third, all dietary records were documented by nutrition experts using photographs provided by participants before and after meals, with actual intake calculated after deducting leftovers. Fourth, we estimated the iodine intake of postpartum women on a weekly basis for each week during the 8-week postpartum period.

Overall, this study does not provide conclusive evidence regarding the long-term thyroid effects of temporary iodine excess during the postpartum period. Given the limited sample size, high attrition, and single time-point assessment of thyroid function, these findings should be interpreted with caution, and potential associations cannot be ruled out. Larger, well-designed longitudinal cohort studies with repeated thyroid measurements are required to more definitively clarify the implications of postpartum iodine exposure.

## 5. Conclusions

In this follow-up study of Korean postpartum women, no cases of thyroid disease were identified one year after childbirth, and no statistically significant differences in thyroid hormone abnormalities (T3, FT4, and TSH) were observed across quartiles of seaweed-derived iodine intake. However, these findings do not provide conclusive evidence regarding the long-term thyroid effects of short-term postpartum iodine excess.

Because of the limited sample size, substantial loss to follow-up, and restriction to a single time-point assessment of thyroid hormones, the study may have been underpowered to detect clinically meaningful associations, and potential risks cannot be ruled out. Accordingly, the results should be interpreted with caution. These findings are specific to healthy Korean postpartum women residing in postpartum care centers and should not be generalized to other postpartum populations or to women with pre-existing thyroid disease. Larger, well-designed longitudinal studies incorporating repeated thyroid measurements and broader thyroid outcomes are required to clarify the implications of postpartum iodine exposure and to better inform dietary guidance during this period.

## Figures and Tables

**Table 1:** Summary of participants’ general characteristics.

Variables	Baseline (N = 147)	Follow-Up (N = 81)
Age (years)	32.8 ± 0.3	33.6 ± 0.5
Height (cm)	161.0 ± 0.8	161.2 ± 0.6
Weight (kg)		
Full-term	71.2 ± 0.9	70.0 ± 1.0
Postpartum	64.5 ± 0.8	63.5 ± 1.0
Health status (%)		
Very healthy	19 (12.9)	12 (14.8)
Healthy	78 (53.1)	42 (51.9)
Normal	50 (34.0)	27 (33.3)
Family history of thyroid disease (%)		
Thyroid cancer	9 (6.1)	9 (11.1)
Hypothyroidism	3 (2.1)	3 (3.7)
Hyperthyroidism	2 (1.4)	1 (1.2)
None	133 (90.5)	68 (84.0)
Frequency of cruciferous food intake during postpartum periods (%)		
Less than once	25 (17.0)	13 (16.1)
1 time	20 (13.6)	9 (11.1)
2 times	44 (29.9)	24 (29.6)
3 times	25 (17.0)	15 (18.5)
4 times	15 (10.2)	8 (9.9)
5 or more times	18 (12.2)	12 (14.8)
Gestation period (week)	38.4 ± 0.2	38.2 ± 0.2
Birth type (%)		
Natural	70 (47.6)	38 (46.9)
Cesarean section	77 (52.4)	43 (53.1)
First-born (%)		
First	84 (57.1)	46 (56.8)
Other	63 (42.9)	35 (43.2)
Premature birth (%)		
Premature	5 (3.4)	1 (1.2)
Full-term	142 (96.6)	80 (98.8)
Lactation type (%)		
Breast milk	140 (95.2)	71 (87.7)
Mixed (Breast + Formula)	7 (4.8)	10 (12.3)
Newborn growth indicators		
Length (cm)	50.1 ± 0.4	78.2 ± 0.5
Weight (kg)	3.3 ± 0.2	10.5 ± 0.1

Data are presented as the mean ± standard error or number (%) of participants. Full-term birth was defined based on gestational age at delivery. Postpartum body weight was measured at admission to the postpartum care center.

**Table 2:** Prevalence of abnormal T3, FT4, and TSH by iodine intake quartiles during the postpartum period.

Variables	Q1 (N = 20)	Q2 (N = 20)	Q3 (N = 21)	Q4 (N = 20)	*p* Value
Intake range (μg/day)	32.4–481.5	483.4–1100.7	1140.6–1718.9	1732.2–3241.1	
Median intake (μg/day)	297.5	721.1	1299.9	2173.1	
T3					
Normal	17 (85.0)	19 (95.0)	16 (76.2)	17 (85.0)	0.4175
Abnormal	3 (15.0)	1 (5.0)	5 (23.8)	3 (15.0)	
FT4					
Normal	19 (95.0)	20 (100)	19 (90.5)	16 (80.0)	0.1591
Abnormal	1 (5.0)	0 (0)	2 (9.5)	4 (20.0)	
TSH					
Normal	19 (95.0)	18 (90.0)	20 (95.2)	19 (95.0)	0.9344
Abnormal	1 (5.0)	2 (10.0)	1 (4.8)	1 (5.0)	

Normal ranges: T3: 0.8–2.0 ng/mL, FT4: 0.93–1.70 pg/dL, TSH: 0.35–5.50 µIU/mL; Q: quartile; categorical variables are reported as numbers (%).

**Table 3:** One-year follow-up prevalence of abnormal T3, FT4, and TSH by iodine intake quartiles.

Variables	Q1 (N = 20)	Q2 (N = 20)	Q3 (N = 21)	Q4 (N = 20)	*p* Value
Intake range (μg/day)	1.7–38.2	38.5–59.1	60.9–135.8	195.9–4607.1	
Median intake (μg/day)	26.5	45.4	70.4	363.4	
T3					
Normal	17 (85.0)	17 (85.0)	17 (81.0)	18 (90.0)	0.9721
Abnormal	3 (15.0)	3 (15.0)	4 (19.1)	2 (10.0)	
FT4					
Normal	19 (95.0)	19 (95.0)	16 (76.2)	20 (100)	0.0589
Abnormal	1 (5.0)	1 (5.0)	5 (23.8)	0 (0)	
TSH					
Normal	19 (95.0)	20 (100)	19 (90.0)	18 (90.0)	0.7476
Abnormal	1 (5.0)	0 (0)	2 (9.5)	2 (10.0)	

Normal ranges: T3: 0.8–2.0 ng/mL, FT4: 0.93–1.70 pg/dL, TSH: 0.35–5.50 µIU/mL; Q: quartile; categorical variables are reported as numbers (%).

**Table 4:** Daily iodine intake and urinary iodine concentration (UIC) of participants at the 1-year follow-up.

	Iodine Intake (μg/Day)	UIC (μg/L)
Mean ± SE	220.5 ± 65.5	423.1 ± 88.2
Range	1.7–4607.1	22.2–5749.7
Median	60.9	194.6

UIC: urinary iodine concentration, measured from a single spot urine sample; according to the WHO, adequate iodine intake among adults corresponds to a median UIC of 100–199 μg/L.

## Data Availability

The dataset supporting the results of this research is openly available at https://figshare.com/articles/dataset/Iodine_Postpartum_Follow-up/29595911?file=56367293 (accessed on 18 July 2025), and access does not require permission.

## Supplementary Materials

The following supporting information can be downloaded at https://www.mdpi.com/article/10.3390/healthcare14030298/s1, Table S1: General characteristics of participants (Continued); Table S2: Comparison of baseline characteristics between retained and withdrawn participants; Table S3: Nutrient intake during the postpartum period and one year after follow-up; Table S4. Total iodine intake and iodine intake from seaweed soup during the postpartum period.
