# Supplementary material for: Seaweed-Derived Iodine Intake During the Korean Postpartum Period: A 1-Year Follow-Up Study
Source: Healthcare (Basel). 2026 Jan 24;14(3):298. doi: 10.3390/healthcare14030298 (PMC12897452; doi:10.3390/healthcare14030298)
Supplement: Supplementary file 1 [file healthcare-14-00298-s001.zip › healthcare-4049551-supplementary.pdf]

Supplementary Table S1. General characteristics of participants (Continued).

	Baseline (N = 147)	Follow-up (N = 81)	P value
Disease			0.98
Asthma	1 (0.7)	1 (1.2)	
Atopy	2 (1.4)	1 (1.2)	
Gastritis	1 (0.7)	1 (1.2)	
Hepatitis B	1 (0.7)	1 (1.2)	
Herniated disc	1 (0.7)	1 (1.2)	
None	141 (95.9)	76 (93.8)	
Smoking			0.98
Non-smoker	125 (85.0)	69 (85.2)	
Ex-smoker	22 (15.0)	12 (14.8)	
Lactating with drinking	0 (0.0)	0 (0.0)	
Exercise			0.34
Non-exercise	122 (83.0)	63 (77.8)	
Exercise	25 (17.0)	18 (22.2)	
Education level			0.75
High school	16 (10.9)	11 (13.6)	
College	115 (78.3)	63 (77.8)	
Graduate school	16 (10.9)	7 (8.6)	
Income level (10,000KRW/month)			0.80
< 100	2 (1.4)	1 (1.2)	
100 ≤ to < 200	9 (6.1)	7 (8.6)	
200 ≤ to < 300	30 (20.4)	15 (18.5)	
300 ≤ to < 400	46 (31.3)	32 (39.5)	

400 ≤ to < 500	21 (14.3)	10 (12.4)
500 ≤ to < 600	18 (12.2)	6 (7.4)
≥ 600	21 (14.3)	10 (12.4)

All data represent mean ± standard error or number (%) of participants.

Formatted: Font: (Default) Times New Roman, Complex Script Font: Times New Roman

Formatted Table

Supplementary Table S2. Comparison of baseline characteristics between retained and withdrawn participants

Variables	Retained participants (N = 81)	Withdrawn participants (N = 66)	P value
Age (years)	33.6 ± 0.5	32.4±0.4	0.761
Height (cm)	161.2 ± 0.6	162.4±0.6	0.105
Weight (kg)			
Full-term	70.0 ± 1.0	72.7±1.4	0.112
Postpartum	63.5 ± 1.0	66.1±1.3	0.108
Health status (%)			0.750
Very healthy	12 (14.8)	7(4.8)	
Healthy	42 (51.9)	36(24.5)	
Normal	27 (33.3)	23(15.6)	
Family history of thyroid disease (%)			0.014
Thyroid cancer	9 (11.1)	0(0)	
Hypothyroidism	3 (3.7)	0(0)	
Hyperthyroidism	1 (1.2)	1(0.7)	
None	68 (84.0)	65(44.2)	
Frequency of cruciferous food intake during postpartum periods (%)			0.878
Less than once	13 (16.1)	12(8.2)	
1 time	9 (11.1)	11(7.5)	
2 times	24 (29.6)	20(13.6)	
3 times	15 (18.5)	10(6.8)	
4 times	8 (9.9)	7(4.8)	
5 or more times	12 (14.8)	6(4.1)	
Gestation period (week)	38.2 ± 0.2	38.2±0.3	0.348
Birth type (%)			0.850
Natural	38 (46.9)	32(21.8)	
Cesarean section	43 (53.1)	34(23.1)	
First-born (%)			0.924
First	46 (56.8)	38(25.9)	
Other	35 (43.2)	28(19)	
Premature birth (%)			0.108
Premature	1 (1.2)	4(2.7)	
Full-term	80 (98.8)	62(42.2)	
Lactation type (%)			0.091
Breast milk	71 (87.7)	58(39.5)	
Mixed (Breast + Formula)	10 (12.3)	8(5.4)	
Newborn growth indicators			
Length (cm)	78.2 ± 0.5	50.5±0.2	0.423
Weight (kg)	10.5 ± 0.1	3.3±0.1	0.476
Disease			0.644
Asthma	1 (1.2)	0(0)	
Atopy	1 (1.2)	1(0.7)	
Gastritis	1 (1.2)	0(0)	
Hepatitis B	1 (1.2)	0(0)	
Herniated disc	1 (1.2)	0(0)	
None	76 (93.8)	65(44.2)	

Formatted ... [4]
Formatted ... [1]
Formatted Table ... [2]
Formatted ... [3]
Formatted ... [5]
Formatted ... [6]
Formatted ... [7]
Formatted ... [8]
Formatted ... [9]
Formatted ... [10]
Formatted ... [11]
Formatted ... [12]
Formatted ... [13]
Formatted ... [14]
Formatted ... [15]
Formatted ... [16]
Formatted ... [17]
Formatted ... [18]
Formatted ... [19]
Formatted ... [20]
Formatted ... [21]
Formatted ... [22]
Formatted ... [23]
Formatted ... [24]
Formatted ... [25]
Formatted ... [26]
Formatted ... [27]
Formatted ... [28]
Formatted ... [29]
Formatted ... [30]
Formatted ... [31]
Formatted ... [32]
Formatted ... [33]
Formatted ... [34]
Formatted ... [35]
Formatted ... [36]
Formatted ... [37]
Formatted ... [38]
Formatted ... [39]
Formatted ... [40]
Formatted ... [41]
Formatted ... [42]
Formatted ... [43]
Formatted ... [44]
Formatted ... [45]
Formatted ... [46]
Formatted ... [47]
Formatted ... [48]
Formatted ... [49]
Formatted ... [50]
Formatted ... [51]
Formatted ... [52]
Formatted ... [53]
Formatted ... [54]
Formatted ... [55]
Formatted ... [56]
Formatted ... [57]

Smoking			0.955
Non-smoker	69 (85.2)	56(38.1)	
Ex-smoker	12 (14.8)	10(6.8)	
Lactating with drinking	0 (0.0)	0 (0.0)	
Exercise			0.326
Non-exercise	63 (77.8)	57(38.8)	
Exercise	18 (22.2)	9(6.1)	
Education level			0.360
High school	11 (13.6)	5(3.4)	
College	63 (77.8)	52(35.4)	
Graduate school	7 (8.6)	9(6.1)	
Income level (10,000KRW/month)			0.105
< 100	1 (1.2)	1(0.7)	
100 ≤ to < 200	7 (8.6)	2(1.4)	
200 ≤ to < 300	15 (18.5)	15(10.2)	
300 ≤ to < 400	32 (39.5)	14(9.5)	
400 ≤ to < 500	10 (12.4)	11(7.5)	
500 ≤ to < 600	6 (7.4)	12(8.2)	
≥ 600	10 (12.4)	11(7.5)	

All data represent mean ± standard error or number (%) of participants; T-test was used for continuous variables.

Formatted ... [96]
Formatted ... [95]
Formatted ... [97]
Formatted ... [98]
Formatted ... [99]
Formatted ... [100]
Formatted ... [101]
Formatted ... [102]
Formatted ... [104]
Formatted ... [105]
Formatted ... [106]
Formatted ... [103]
Formatted ... [108]
Formatted ... [107]
Formatted ... [109]
Formatted ... [110]
Formatted ... [111]
Formatted ... [112]
Formatted ... [113]
Formatted ... [114]
Formatted ... [116]
Formatted ... [117]
Formatted ... [115]
Formatted ... [118]
Formatted ... [119]
Formatted ... [120]
Formatted ... [121]
Formatted ... [122]
Formatted ... [123]
Formatted ... [124]
Formatted ... [125]
Formatted ... [126]
Formatted ... [128]
Formatted ... [127]
Formatted ... [129]
Formatted ... [130]
Formatted ... [131]
Formatted ... [132]
Formatted ... [133]
Formatted ... [134]
Formatted ... [135]
Formatted ... [136]
Formatted ... [137]
Formatted ... [138]
Formatted ... [139]
Formatted ... [140]
Formatted ... [141]
Formatted ... [142]
Formatted ... [143]
Formatted ... [144]
Formatted ... [145]
Formatted ... [146]
Formatted ... [147]
Formatted ... [148]
Formatted ... [149]

Supplementary Table S32. Nutrient intake during the postpartum period and one year after follow-up.

Nutrients intake per day	Entire postpartum period (N = 81)	One year after childbirth (N = 81)	P value
Energy (kcal)	1847.0 ± 45.1	1543.2 ± 50.8	<.0001
Carbohydrate (g)	237.0 ± 6.1	197.1 ± 6.2	<.0001
Fat (g)	61.5 ± 1.5	53.0 ± 2.6	0.0056
Protein (g)	75.2 ± 1.9	59.9 ± 2.2	<.0001
Fiber (g)	384.7 ± 14.7	14.6 ± 0.7	<.0001
Water (g)	697.6 ± 22.1	653.6 ± 29.1	0.2294
Vitamin A (μg RAE)	450.9 ± 20.4	346.9 ± 24.2	0.0013
Retinol (μg)	139.1 ± 6.8	140.6 ± 14.8	0.9288
β-carotene (μg)	3741.3 ± 211.3	2475.6 ± 224.1	<.0001
Vitamin D (μg)	3.8 ± 0.2	3.0 ± 0.3	0.0443
Vitamin E (mg)	21.8 ± 0.8	15.1 ± 0.7	<.0001
Vitamin K (μg)	216.4 ± 15.4	96.7 ± 7.1	<.0001
Vitamin C (mg)	70.7 ± 4.0	43.0 ± 3.1	<.0001
Thiamine (mg)	1.6 ± 0.05	1.3 ± 0.1	<.0001
Riboflavin (mg)	1.5 ± 0.05	1.2 ± 0.1	0.0002
Niacin (mg)	13.2 ± 0.3	10.4 ± 0.4	<.0001
Vitamin B_6 (mg)	1.9 ± 0.09	1.4 ± 0.1	0.0038
Folic acid (μg)	424.7 ± 16.6	299.8 ± 12.0	<.0001
Vitamin B_{12} (μg)	9.7 ± 0.4	6.5 ± 0.4	<.0001
Calcium (mg)	517.2 ± 18.2	408.5 ± 18.5	<.0001
Phosphorus (mg)	1104.4 ± 30.0	854.3 ± 30.9	<.0001

Sodium (mg)	4452.7 ± 171.1	2958.1 ± 116.1	<.0001
Potassium (mg)	2672.0 ± 92.2	1822.5 ± 73.8	<.0001
Magnesium (mg)	133.9 ± 5.6	73.8 ± 3.9	<.0001
Iron (mg)	16.1 ± 0.5	11.2 ± 0.4	<.0001
Copper (µg)	687.6 ± 28.4	518.1 ± 28.6	<.0001
Iodine (µg)	1159.0 ± 86.2	220.5 ± 65.5	<.0001

Continuous variables are reported as mean ± standard error; T-test was used for continuous variables.

Formatted Table

Supplementary Table S4~~3~~. Total iodine intake and iodine intake from seaweed soup during the postpartum period.

	Total iodine intake (µg/day) (N = 147)	Iodine intake from seaweed soup (µg/day) (N = 147)
week 1	2455.6 ± 175.3	2356.12 ± 172.2
week 2	1800.9 ± 180.4	1631.7 ± 170.3
week 3	1252.7 ± 140.8	1173.6 ± 138.7
week 4	966.2 ± 108.7	836.2 ± 103.9
week 5	687.8 ± 83.8	626.3 ± 84.3
week 6	868.9 ± 150.9	660.1 ± 120.4
week 7	672.4 ± 101.7	478.6 ± 74.9
week 8	567.1 ± 64.3	473.7 ± 63.3
Entire postpartum period	1159.0 ± 86.2	1029.6 ± 85.0

Iodine intake was presented as mean ± standard error through ANOVA; Scheffe's post hoc tests are used for differences between means.

Formatted Table

Formatted Table

Formatted: Font: (Default) Times New Roman, Complex Script Font: Times New Roman

Page 3: [1] Formatted	Jihee Choi	12/25/2025 12:46:00 PM

Font: Times New Roman, 11 pt, Complex Script Font: 11 pt

Page 3: [2] Formatted Table	Jihee Choi	12/25/2025 12:38:00 PM

Formatted Table

Page 3: [3] Formatted	Jihee Choi	12/25/2025 12:46:00 PM

Font: Times New Roman, 11 pt, Complex Script Font: 11 pt

Page 3: [4] Formatted	Jihee Choi	12/25/2025 12:37:00 PM

Centered

Page 3: [5] Formatted	Jihee Choi	12/25/2025 12:46:00 PM

Font: Times New Roman, 11 pt, Complex Script Font: 11 pt

Page 3: [6] Formatted	Jihee Choi	12/25/2025 12:46:00 PM

Font: Times New Roman

Page 3: [6] Formatted	Jihee Choi	12/25/2025 12:46:00 PM

Font: Times New Roman

Page 3: [7] Formatted	Jihee Choi	12/25/2025 12:46:00 PM

Font: Times New Roman, 11 pt, Complex Script Font: 11 pt

Page 3: [8] Formatted **Jihee Choi** **12/25/2025 12:46:00 PM**

Font: Times New Roman

Page 3: [8] Formatted **Jihee Choi** **12/25/2025 12:46:00 PM**

Font: Times New Roman

Page 3: [9] Formatted **Jihee Choi** **12/25/2025 12:46:00 PM**

Font: Times New Roman, 11 pt, Complex Script Font: 11 pt

Page 3: [10] Formatted **Jihee Choi** **12/25/2025 12:46:00 PM**

Font: Times New Roman, 11 pt, Complex Script Font: 11 pt

Page 3: [11] Formatted **Jihee Choi** **12/25/2025 12:46:00 PM**

Font: Times New Roman

Page 3: [11] Formatted **Jihee Choi** **12/25/2025 12:46:00 PM**

Font: Times New Roman

Page 3: [12] Formatted **Jihee Choi** **12/25/2025 12:46:00 PM**

Font: Times New Roman

Page 3: [13] Formatted **Jihee Choi** **12/25/2025 12:46:00 PM**

Font: Times New Roman, 11 pt, Complex Script Font: 11 pt

Page 3: [14] Formatted	Jihee Choi	12/25/2025 12:46:00 PM

Font: Times New Roman

Page 3: [14] Formatted	Jihee Choi	12/25/2025 12:46:00 PM

Font: Times New Roman

Page 3: [15] Formatted	Jihee Choi	12/25/2025 12:46:00 PM

Font: Times New Roman

Page 3: [16] Formatted	Jihee Choi	12/25/2025 12:46:00 PM

Font: Times New Roman, 11 pt, Complex Script Font: 11 pt

Page 3: [17] Formatted	Jihee Choi	12/25/2025 12:46:00 PM

Font: Times New Roman, 11 pt, Complex Script Font: 11 pt

Page 3: [18] Formatted	Jihee Choi	12/25/2025 12:46:00 PM

Font: Times New Roman

Page 3: [18] Formatted	Jihee Choi	12/25/2025 12:46:00 PM

Font: Times New Roman

Page 3: [19] Formatted	Jihee Choi	12/25/2025 12:46:00 PM

Font: Times New Roman, 11 pt, Complex Script Font: 11 pt

Page 3: [20] Formatted **Jihee Choi** **12/25/2025 12:46:00 PM**

Font: Times New Roman

Page 3: [20] Formatted **Jihee Choi** **12/25/2025 12:46:00 PM**

Font: Times New Roman

Page 3: [21] Formatted **Jihee Choi** **12/25/2025 12:46:00 PM**

Font: Times New Roman, 11 pt, Complex Script Font: 11 pt

Page 3: [22] Formatted **Jihee Choi** **12/25/2025 12:46:00 PM**

Font: Times New Roman

Page 3: [22] Formatted **Jihee Choi** **12/25/2025 12:46:00 PM**

Font: Times New Roman

Page 3: [23] Formatted **Jihee Choi** **12/25/2025 12:46:00 PM**

Font: Times New Roman, 11 pt, Complex Script Font: 11 pt

Page 3: [24] Formatted **Jihee Choi** **12/25/2025 12:46:00 PM**

Font: Times New Roman, 11 pt, Complex Script Font: 11 pt

Page 3: [25] Formatted **Jihee Choi** **12/25/2025 12:46:00 PM**

Font: Times New Roman

Page 3: [25] Formatted	Jihee Choi	12/25/2025 12:46:00 PM

Font: Times New Roman

Page 3: [26] Formatted	Jihee Choi	12/25/2025 12:46:00 PM

Font: Times New Roman, 11 pt, Complex Script Font: 11 pt

Page 3: [27] Formatted	Jihee Choi	12/25/2025 12:46:00 PM

Font: Times New Roman

Page 3: [27] Formatted	Jihee Choi	12/25/2025 12:46:00 PM

Font: Times New Roman

Page 3: [28] Formatted	Jihee Choi	12/25/2025 12:46:00 PM

Font: Times New Roman, 11 pt, Complex Script Font: 11 pt

Page 3: [29] Formatted	Jihee Choi	12/25/2025 12:46:00 PM

Font: Times New Roman

Page 3: [29] Formatted	Jihee Choi	12/25/2025 12:46:00 PM

Font: Times New Roman

Page 3: [30] Formatted	Jihee Choi	12/25/2025 12:46:00 PM

Font: Times New Roman, 11 pt, Complex Script Font: 11 pt

Page 3: [31] Formatted	Jihee Choi	12/25/2025 12:46:00 PM

Font: Times New Roman

Page 3: [31] Formatted	Jihee Choi	12/25/2025 12:46:00 PM

Font: Times New Roman

Page 3: [32] Formatted	Jihee Choi	12/25/2025 12:46:00 PM

Font: Times New Roman, 11 pt, Complex Script Font: 11 pt

Page 3: [33] Formatted	Jihee Choi	12/25/2025 12:46:00 PM

Font: Times New Roman, 11 pt, Complex Script Font: 11 pt

Page 3: [34] Formatted	Jihee Choi	12/25/2025 12:46:00 PM

Font: Times New Roman

Page 3: [34] Formatted	Jihee Choi	12/25/2025 12:46:00 PM

Font: Times New Roman

Page 3: [35] Formatted	Jihee Choi	12/25/2025 12:46:00 PM

Font: Times New Roman, 11 pt, Complex Script Font: 11 pt

Page 3: [36] Formatted	Jihee Choi	12/25/2025 12:46:00 PM

Font: Times New Roman

Page 3: [36] Formatted **Jihee Choi** **12/25/2025 12:46:00 PM**

Font: Times New Roman

Page 3: [37] Formatted **Jihee Choi** **12/25/2025 12:46:00 PM**

Font: Times New Roman, 11 pt, Complex Script Font: 11 pt

Page 3: [38] Formatted **Jihee Choi** **12/25/2025 12:46:00 PM**

Font: Times New Roman

Page 3: [38] Formatted **Jihee Choi** **12/25/2025 12:46:00 PM**

Font: Times New Roman

Page 3: [39] Formatted **Jihee Choi** **12/25/2025 12:46:00 PM**

Font: Times New Roman, 11 pt, Complex Script Font: 11 pt

Page 3: [40] Formatted **Jihee Choi** **12/25/2025 12:46:00 PM**

Font: Times New Roman

Page 3: [40] Formatted **Jihee Choi** **12/25/2025 12:46:00 PM**

Font: Times New Roman

Page 3: [41] Formatted **Jihee Choi** **12/25/2025 12:46:00 PM**

Font: Times New Roman, 11 pt, Complex Script Font: 11 pt

Page 3: [42] Formatted **Jihee Choi** **12/25/2025 12:46:00 PM**

Font: Times New Roman

Page 3: [42] Formatted **Jihee Choi** **12/25/2025 12:46:00 PM**

Font: Times New Roman

Page 3: [43] Formatted **Jihee Choi** **12/25/2025 12:46:00 PM**

Font: Times New Roman, 11 pt, Complex Script Font: 11 pt

Page 3: [44] Formatted **Jihee Choi** **12/25/2025 12:46:00 PM**

Font: Times New Roman

Page 3: [44] Formatted **Jihee Choi** **12/25/2025 12:46:00 PM**

Font: Times New Roman

Page 3: [45] Formatted **Jihee Choi** **12/25/2025 12:46:00 PM**

Font: Times New Roman, 11 pt, Complex Script Font: 11 pt

Page 3: [46] Formatted **Jihee Choi** **12/25/2025 12:46:00 PM**

Font: Times New Roman

Page 3: [46] Formatted **Jihee Choi** **12/25/2025 12:46:00 PM**

Font: Times New Roman

Page 3: [47] Formatted	Jihee Choi	12/25/2025 12:46:00 PM

Font: Times New Roman

Page 3: [48] Formatted	Jihee Choi	12/25/2025 12:46:00 PM

Font: Times New Roman, 11 pt, Complex Script Font: 11 pt

Page 3: [49] Formatted	Jihee Choi	12/25/2025 12:46:00 PM

Font: Times New Roman, 11 pt, Complex Script Font: 11 pt

Page 3: [50] Formatted	Jihee Choi	12/25/2025 12:46:00 PM

Font: Times New Roman

Page 3: [50] Formatted	Jihee Choi	12/25/2025 12:46:00 PM

Font: Times New Roman

Page 3: [51] Formatted	Jihee Choi	12/25/2025 12:46:00 PM

Font: Times New Roman, 11 pt, Complex Script Font: 11 pt

Page 3: [52] Formatted	Jihee Choi	12/25/2025 12:46:00 PM

Font: Times New Roman

Page 3: [52] Formatted	Jihee Choi	12/25/2025 12:46:00 PM

Font: Times New Roman

Page 3: [53] Formatted	Jihee Choi	12/25/2025 12:46:00 PM

Font: Times New Roman, 11 pt, Complex Script Font: 11 pt

Page 3: [54] Formatted	Jihee Choi	12/25/2025 12:46:00 PM

Font: Times New Roman, 11 pt, Complex Script Font: 11 pt

Page 3: [55] Formatted	Jihee Choi	12/25/2025 12:46:00 PM

Font: Times New Roman

Page 3: [55] Formatted	Jihee Choi	12/25/2025 12:46:00 PM

Font: Times New Roman

Page 3: [56] Formatted	Jihee Choi	12/25/2025 12:46:00 PM

Font: Times New Roman, 11 pt, Complex Script Font: 11 pt

Page 3: [57] Formatted	Jihee Choi	12/25/2025 12:46:00 PM

Font: Times New Roman

Page 3: [57] Formatted	Jihee Choi	12/25/2025 12:46:00 PM

Font: Times New Roman

Page 3: [58] Formatted	Jihee Choi	12/25/2025 12:46:00 PM

Font: Times New Roman, 11 pt, Complex Script Font: 11 pt

Page 3: [59] Formatted **Jihee Choi** **12/25/2025 12:46:00 PM**

Font: Times New Roman, 11 pt, Complex Script Font: 11 pt

Page 3: [60] Formatted **Jihee Choi** **12/25/2025 12:46:00 PM**

Font: Times New Roman

Page 3: [60] Formatted **Jihee Choi** **12/25/2025 12:46:00 PM**

Font: Times New Roman

Page 3: [61] Formatted **Jihee Choi** **12/25/2025 12:46:00 PM**

Font: Times New Roman, 11 pt, Complex Script Font: 11 pt

Page 3: [62] Formatted **Jihee Choi** **12/25/2025 12:46:00 PM**

Font: Times New Roman

Page 3: [62] Formatted **Jihee Choi** **12/25/2025 12:46:00 PM**

Font: Times New Roman

Page 3: [63] Formatted **Jihee Choi** **12/25/2025 12:46:00 PM**

Font: Times New Roman, 11 pt, Complex Script Font: 11 pt

Page 3: [64] Formatted **Jihee Choi** **12/25/2025 12:46:00 PM**

Font: Times New Roman, 11 pt, Complex Script Font: 11 pt

Page 3: [65] Formatted **Jihee Choi** **12/25/2025 12:46:00 PM**

Font: Times New Roman

Page 3: [65] Formatted **Jihee Choi** **12/25/2025 12:46:00 PM**

Font: Times New Roman

Page 3: [66] Formatted **Jihee Choi** **12/25/2025 12:46:00 PM**

Font: Times New Roman, 11 pt, Complex Script Font: 11 pt

Page 3: [67] Formatted **Jihee Choi** **12/25/2025 12:46:00 PM**

Font: Times New Roman

Page 3: [67] Formatted **Jihee Choi** **12/25/2025 12:46:00 PM**

Font: Times New Roman

Page 3: [68] Formatted **Jihee Choi** **12/25/2025 12:46:00 PM**

Font: Times New Roman, 11 pt, Complex Script Font: 11 pt

Page 3: [69] Formatted **Jihee Choi** **12/25/2025 12:46:00 PM**

Font: Times New Roman, 11 pt, Complex Script Font: 11 pt

Page 3: [70] Formatted **Jihee Choi** **12/25/2025 12:46:00 PM**

Font: Times New Roman

Page 3: [70] Formatted **Jihee Choi** **12/25/2025 12:46:00 PM**

Font: Times New Roman

Page 3: [71] Formatted **Jihee Choi** **12/25/2025 12:46:00 PM**

Font: Times New Roman

Page 3: [72] Formatted **Jihee Choi** **12/25/2025 12:46:00 PM**

Font: Times New Roman, 11 pt, Complex Script Font: 11 pt

Page 3: [73] Formatted **Jihee Choi** **12/25/2025 12:46:00 PM**

Font: Times New Roman

Page 3: [73] Formatted **Jihee Choi** **12/25/2025 12:46:00 PM**

Font: Times New Roman

Page 3: [74] Formatted **Jihee Choi** **12/25/2025 12:46:00 PM**

Font: Times New Roman

Page 3: [75] Formatted **Jihee Choi** **12/25/2025 12:27:00 PM**

Indent: Left 0 ch

Page 3: [76] Formatted **Jihee Choi** **12/25/2025 12:46:00 PM**

Font: Times New Roman, 11 pt, Complex Script Font: 11 pt

Page 3: [77] Formatted **Jihee Choi** **12/25/2025 12:46:00 PM**

Font: Times New Roman, 11 pt, Complex Script Font: 11 pt

Page 3: [78] Formatted **Jihee Choi** **12/25/2025 12:46:00 PM**

Font: Times New Roman, 11 pt, Complex Script Font: 11 pt

Page 3: [79] Formatted **Jihee Choi** **12/25/2025 12:46:00 PM**

Font: Times New Roman

Page 3: [79] Formatted **Jihee Choi** **12/25/2025 12:46:00 PM**

Font: Times New Roman

Page 3: [80] Formatted **Jihee Choi** **12/25/2025 12:46:00 PM**

Font: Times New Roman, 11 pt, Complex Script Font: 11 pt

Page 3: [81] Formatted **Jihee Choi** **12/25/2025 12:46:00 PM**

Font: Times New Roman, 11 pt, Complex Script Font: 11 pt

Page 3: [82] Formatted **Jihee Choi** **12/25/2025 12:46:00 PM**

Font: Times New Roman

Page 3: [82] Formatted **Jihee Choi** **12/25/2025 12:46:00 PM**

Font: Times New Roman

Page 3: [83] Formatted **Jihee Choi** **12/25/2025 12:46:00 PM**

Font: Times New Roman, 11 pt, Complex Script Font: 11 pt

Page 3: [84] Formatted **Jihee Choi** **12/25/2025 12:46:00 PM**

Font: Times New Roman, 11 pt, Complex Script Font: 11 pt

Page 3: [85] Formatted **Jihee Choi** **12/25/2025 12:46:00 PM**

Font: Times New Roman

Page 3: [85] Formatted **Jihee Choi** **12/25/2025 12:46:00 PM**

Font: Times New Roman

Page 3: [86] Formatted **Jihee Choi** **12/25/2025 12:46:00 PM**

Font: Times New Roman, 11 pt, Complex Script Font: 11 pt

Page 3: [87] Formatted **Jihee Choi** **12/25/2025 12:46:00 PM**

Font: Times New Roman, 11 pt, Complex Script Font: 11 pt

Page 3: [88] Formatted **Jihee Choi** **12/25/2025 12:46:00 PM**

Font: Times New Roman

Page 3: [88] Formatted **Jihee Choi** **12/25/2025 12:46:00 PM**

Font: Times New Roman

Page 3: [89] Formatted	Jihee Choi	12/25/2025 12:46:00 PM

Font: Times New Roman, 11 pt, Complex Script Font: 11 pt

Page 3: [90] Formatted	Jihee Choi	12/25/2025 12:46:00 PM

Font: Times New Roman, 11 pt, Complex Script Font: 11 pt

Page 3: [91] Formatted	Jihee Choi	12/25/2025 12:46:00 PM

Font: Times New Roman

Page 3: [91] Formatted	Jihee Choi	12/25/2025 12:46:00 PM

Font: Times New Roman

Page 3: [92] Formatted	Jihee Choi	12/25/2025 12:46:00 PM

Font: Times New Roman, 11 pt, Complex Script Font: 11 pt

Page 3: [93] Formatted	Jihee Choi	12/25/2025 12:46:00 PM

Font: Times New Roman, 11 pt, Complex Script Font: 11 pt

Page 3: [94] Formatted	Jihee Choi	12/25/2025 12:46:00 PM

Font: Times New Roman

Page 3: [94] Formatted	Jihee Choi	12/25/2025 12:46:00 PM

Font: Times New Roman

Page 4: [95] Formatted **Jihee Choi** **12/25/2025 12:28:00 PM**

Indent: Left 0 ch

Page 4: [96] Formatted **Jihee Choi** **12/25/2025 12:46:00 PM**

Font: Times New Roman, 11 pt, Complex Script Font: 11 pt

Page 4: [97] Formatted **Jihee Choi** **12/25/2025 12:46:00 PM**

Font: Times New Roman, 11 pt, Complex Script Font: 11 pt

Page 4: [98] Formatted **Jihee Choi** **12/25/2025 12:46:00 PM**

Font: Times New Roman, 11 pt, Complex Script Font: 11 pt

Page 4: [99] Formatted **Jihee Choi** **12/25/2025 12:46:00 PM**

Font: Times New Roman

Page 4: [99] Formatted **Jihee Choi** **12/25/2025 12:46:00 PM**

Font: Times New Roman

Page 4: [100] Formatted **Jihee Choi** **12/25/2025 12:46:00 PM**

Font: Times New Roman, 11 pt, Complex Script Font: 11 pt

Page 4: [101] Formatted **Jihee Choi** **12/25/2025 12:46:00 PM**

Font: Times New Roman, 11 pt, Complex Script Font: 11 pt

Page 4: [102] Formatted **Jihee Choi** **12/25/2025 12:46:00 PM**

Font: Times New Roman

Page 4: [102] Formatted **Jihee Choi** **12/25/2025 12:46:00 PM**

Font: Times New Roman

Page 4: [103] Formatted **Jihee Choi** **12/25/2025 12:28:00 PM**

Indent: Left 0 ch

Page 4: [104] Formatted **Jihee Choi** **12/25/2025 12:46:00 PM**

Font: Times New Roman, 11 pt, Complex Script Font: 11 pt

Page 4: [105] Formatted **Jihee Choi** **12/25/2025 12:46:00 PM**

Font: Times New Roman, 11 pt, Complex Script Font: 11 pt

Page 4: [106] Formatted **Jihee Choi** **12/25/2025 12:46:00 PM**

Font: Times New Roman, 11 pt, Complex Script Font: 11 pt

Page 4: [107] Formatted **Jihee Choi** **12/25/2025 12:28:00 PM**

Indent: Left 0 ch

Page 4: [108] Formatted **Jihee Choi** **12/25/2025 12:46:00 PM**

Font: Times New Roman, 11 pt, Complex Script Font: 11 pt

Page 4: [109] Formatted **Jihee Choi** **12/25/2025 12:46:00 PM**

Font: Times New Roman, 11 pt, Complex Script Font: 11 pt

Page 4: [110] Formatted **Jihee Choi** **12/25/2025 12:46:00 PM**

Font: Times New Roman, 11 pt, Complex Script Font: 11 pt

Page 4: [111] Formatted **Jihee Choi** **12/25/2025 12:46:00 PM**

Font: Times New Roman

Page 4: [111] Formatted **Jihee Choi** **12/25/2025 12:46:00 PM**

Font: Times New Roman

Page 4: [112] Formatted **Jihee Choi** **12/25/2025 12:46:00 PM**

Font: Times New Roman, 11 pt, Complex Script Font: 11 pt

Page 4: [113] Formatted **Jihee Choi** **12/25/2025 12:46:00 PM**

Font: Times New Roman, 11 pt, Complex Script Font: 11 pt

Page 4: [114] Formatted **Jihee Choi** **12/25/2025 12:46:00 PM**

Font: Times New Roman

Page 4: [114] Formatted **Jihee Choi** **12/25/2025 12:46:00 PM**

Font: Times New Roman

Page 4: [115] Formatted **Jihee Choi** **12/25/2025 12:28:00 PM**

Indent: Left 0 ch

Page 4: [116] Formatted **Jihee Choi** **12/25/2025 12:46:00 PM**

Font: Times New Roman, 11 pt, Complex Script Font: 11 pt

Page 4: [117] Formatted **Jihee Choi** **12/25/2025 12:46:00 PM**

Font: Times New Roman

Page 4: [118] Formatted **Jihee Choi** **12/25/2025 12:46:00 PM**

Font: Times New Roman, 11 pt, Complex Script Font: 11 pt

Page 4: [119] Formatted **Jihee Choi** **12/25/2025 12:46:00 PM**

Font: Times New Roman, 11 pt, Complex Script Font: 11 pt

Page 4: [120] Formatted **Jihee Choi** **12/25/2025 12:46:00 PM**

Font: Times New Roman

Page 4: [120] Formatted **Jihee Choi** **12/25/2025 12:46:00 PM**

Font: Times New Roman

Page 4: [121] Formatted **Jihee Choi** **12/25/2025 12:46:00 PM**

Font: Times New Roman, 11 pt, Complex Script Font: 11 pt

Page 4: [122] Formatted **Jihee Choi** **12/25/2025 12:46:00 PM**

Font: Times New Roman, 11 pt, Complex Script Font: 11 pt

Page 4: [123] Formatted **Jihee Choi** **12/25/2025 12:46:00 PM**

Font: Times New Roman

Page 4: [123] Formatted **Jihee Choi** **12/25/2025 12:46:00 PM**

Font: Times New Roman

Page 4: [124] Formatted **Jihee Choi** **12/25/2025 12:46:00 PM**

Font: Times New Roman, 11 pt, Complex Script Font: 11 pt

Page 4: [125] Formatted **Jihee Choi** **12/25/2025 12:46:00 PM**

Font: Times New Roman, 11 pt, Complex Script Font: 11 pt

Page 4: [126] Formatted **Jihee Choi** **12/25/2025 12:46:00 PM**

Font: Times New Roman

Page 4: [126] Formatted **Jihee Choi** **12/25/2025 12:46:00 PM**

Font: Times New Roman

Page 4: [127] Formatted **Jihee Choi** **12/25/2025 12:28:00 PM**

Indent: Left 0 ch

Page 4: [128] Formatted **Jihee Choi** **12/25/2025 12:46:00 PM**

Font: Times New Roman, 11 pt, Complex Script Font: 11 pt

Page 4: [129] Formatted **Jihee Choi** **12/25/2025 12:46:00 PM**

Font: Times New Roman, 11 pt, Complex Script Font: 11 pt

Page 4: [130] Formatted **Jihee Choi** **12/25/2025 12:46:00 PM**

Font: Times New Roman, 11 pt, Complex Script Font: 11 pt

Page 4: [131] Formatted **Jihee Choi** **12/25/2025 12:46:00 PM**

Font: Times New Roman

Page 4: [131] Formatted **Jihee Choi** **12/25/2025 12:46:00 PM**

Font: Times New Roman

Page 4: [132] Formatted **Jihee Choi** **12/25/2025 12:46:00 PM**

Font: Times New Roman, 11 pt, Complex Script Font: 11 pt

Page 4: [133] Formatted **Jihee Choi** **12/25/2025 12:46:00 PM**

Font: Times New Roman, 11 pt, Complex Script Font: 11 pt

Page 4: [134] Formatted **Jihee Choi** **12/25/2025 12:46:00 PM**

Font: Times New Roman

Page 4: [134] Formatted **Jihee Choi** **12/25/2025 12:46:00 PM**

Font: Times New Roman

Page 4: [135] Formatted **Jihee Choi** **12/25/2025 12:46:00 PM**

Font: Times New Roman, 11 pt, Complex Script Font: 11 pt

Page 4: [136] Formatted **Jihee Choi** **12/25/2025 12:46:00 PM**

Font: Times New Roman, 11 pt, Complex Script Font: 11 pt

Page 4: [137] Formatted **Jihee Choi** **12/25/2025 12:46:00 PM**

Font: Times New Roman

Page 4: [137] Formatted **Jihee Choi** **12/25/2025 12:46:00 PM**

Font: Times New Roman

Page 4: [138] Formatted **Jihee Choi** **12/25/2025 12:46:00 PM**

Font: Times New Roman, 11 pt, Complex Script Font: 11 pt

Page 4: [139] Formatted **Jihee Choi** **12/25/2025 12:46:00 PM**

Font: Times New Roman, 11 pt, Complex Script Font: 11 pt

Page 4: [140] Formatted **Jihee Choi** **12/25/2025 12:46:00 PM**

Font: Times New Roman

Page 4: [140] Formatted **Jihee Choi** **12/25/2025 12:46:00 PM**

Font: Times New Roman

Page 4: [141] Formatted **Jihee Choi** **12/25/2025 12:46:00 PM**

Font: Times New Roman, 11 pt, Complex Script Font: 11 pt

Page 4: [142] Formatted **Jihee Choi** **12/25/2025 12:46:00 PM**

Font: Times New Roman, 11 pt, Complex Script Font: 11 pt

Page 4: [143] Formatted **Jihee Choi** **12/25/2025 12:46:00 PM**

Font: Times New Roman

Page 4: [143] Formatted **Jihee Choi** **12/25/2025 12:46:00 PM**

Font: Times New Roman

Page 4: [144] Formatted **Jihee Choi** **12/25/2025 12:46:00 PM**

Font: Times New Roman, 11 pt, Complex Script Font: 11 pt

Page 4: [145] Formatted **Jihee Choi** **12/25/2025 12:46:00 PM**

Font: Times New Roman, 11 pt, Complex Script Font: 11 pt

Page 4: [146] Formatted **Jihee Choi** **12/25/2025 12:46:00 PM**

Font: Times New Roman

Page 4: [146] Formatted	Jihee Choi	12/25/2025 12:46:00 PM

Font: Times New Roman

Page 4: [147] Formatted	Jihee Choi	12/25/2025 12:46:00 PM

Font: Times New Roman, 11 pt, Complex Script Font: 11 pt

Page 4: [148] Formatted	Jihee Choi	12/25/2025 12:46:00 PM

Font: Times New Roman, 11 pt, Complex Script Font: 11 pt

Page 4: [149] Formatted	Jihee Choi	12/25/2025 12:46:00 PM

Font: Times New Roman

Page 4: [149] Formatted	Jihee Choi	12/25/2025 12:46:00 PM

Font: Times New Roman
